# Supplementary material for: Curated and harmonised transcriptomics datasets of interstitial lung diseases
Source: Data Brief. 2025 Oct 14;63:112139. doi: 10.1016/j.dib.2025.112139 (PMC12581653; doi:10.1016/j.dib.2025.112139)
Supplement: Supplementary file 1 [file mmc1.zip › Supplementary_material/RNA-seq/fastQC reports/GSE180415/TRIMMED_SRR15194702_1_fastqc.html]

TRIMMED\_SRR15194702\_1.fastq.gz FastQC Report 

FastQC Report

Wed 25 Oct 2023  
TRIMMED\_SRR15194702\_1.fastq.gz

## Summary

- Basic Statistics
- Per base sequence quality
- Per sequence quality scores
- Per base sequence content
- Per sequence GC content
- Per base N content
- Sequence Length Distribution
- Sequence Duplication Levels
- Overrepresented sequences
- Adapter Content

## Basic Statistics

| Measure | Value |
| --- | --- |
| Filename | TRIMMED\_SRR15194702\_1.fastq.gz |
| File type | Conventional base calls |
| Encoding | Sanger / Illumina 1.9 |
| Total Sequences | 41427708 |
| Sequences flagged as poor quality | 0 |
| Sequence length | 60-80 |
| %GC | 53 |

## Per base sequence quality

## Per sequence quality scores

## Per base sequence content

## Per sequence GC content

## Per base N content

## Sequence Length Distribution

## Sequence Duplication Levels

## Overrepresented sequences

| Sequence | Count | Percentage | Possible Source |
| --- | --- | --- | --- |
| CTCAGACCGCGTTCTCTCCCTCTCACTCCCCAATACGGAGAGAAGAACGA | 131330 | 0.31701005520266773 | No Hit |
| CCAGGCTGGAGTGCAGTGGCTATTCACAGGCGCGATCCCACTACTGATCA | 61437 | 0.1482992976584657 | No Hit |
| GGGCGGGCTCCCGGCCCCGGCCGACGCGCCGCGAGGCGAGCCGGGCGGGC | 54317 | 0.1311127325701919 | No Hit |
| CTGGAGTCTTGGAAGCTTGACTACCCTACGTTCTCCTACAAATGGACCTT | 52561 | 0.12687402353999405 | No Hit |

## Adapter Content

Produced by FastQC (version 0.11.7)
